# Culture-Dependent and -Independent Identification of Polyphosphate-Accumulating *Dechloromonas* spp. Predominating in a Full-Scale Oxidation Ditch Wastewater Treatment Plant

**DOI:** 10.1264/jsme2.ME16097

**Published:** 2016-11-19

**Authors:** Mia Terashima, Ayano Yama, Megumi Sato, Isao Yumoto, Yoichi Kamagata, Souichiro Kato

**Affiliations:** 1 Bioproduction Research Institute, National Institute of Advanced Industrial Science and Technology (AIST)2–17–2–1 Tsukisamu-Higashi, Toyohira-ku, Sapporo, Hokkaido 062–8517Japan; 2 Division of Applied Bioscience, Graduate School of Agriculture, Hokkaido UniversityKita-9 Nishi-9, Kita-ku, Sapporo, Hokkaido 060–8589Japan; 3 Hokkaido High-Technology College2–12–1 Megumino-kita, Eniwa, Hokkaido 061–1374Japan

**Keywords:** wastewater treatment, oxidation ditch, biological phosphorus removal, *Dechloromonas* sp., polyphosphate accumulation

## Abstract

The oxidation ditch process is one of the most economical approaches currently used to simultaneously remove organic carbon, nitrogen, and also phosphorus (P) from wastewater. However, limited information is available on biological P removal in this process. In the present study, microorganisms contributing to P removal in a full-scale oxidation ditch reactor were investigated using culture-dependent and -independent approaches. A microbial community analysis based on 16S rRNA gene sequencing revealed that a phylotype closely related to *Dechloromonas* spp. in the family *Rhodocyclaceae* dominated in the oxidation ditch reactor. This dominant *Dechloromonas* sp. was successfully isolated and subjected to fluorescent staining for polyphosphate, followed by microscopic observations and a spectrofluorometric analysis, which clearly demonstrated that the *Dechloromonas* isolate exhibited a strong ability to accumulate polyphosphate within its cells. These results indicate the potential key role of *Dechloromonas* spp. in efficient P removal in the oxidation ditch wastewater treatment process.

Nutrients such as organic carbon (C), nitrogen (N), and phosphorus (P) have to be removed from sewage/wastewater to avoid the eutrophication of aquatic water systems. Diverse types of wastewater treatment plants (WWTPs) that rely on microbial metabolic activities have been developed and utilized to achieve the efficient removal of nutrients. Aerobic treatment processes, such as activated sludge plants, are commonly utilized as sewage treatments. Aerobic processes are generally efficient for C removal, but not for N or P removal. Since the biological removal of N and P involves aerobic and anaerobic metabolism, conventional aerobic processes require additional reactors with different dissolved oxygen concentrations. The simultaneous removal of C, N, and P may be achieved in one reactor by alternating between aerobic and anaerobic phases. These processes are often referred to as simultaneous nitrification, denitrification, and phosphate removal (SNDPR) (54, 55).

Microbial metabolism related to N removal in WWTPs has been extensively examined (35). Ammonia-N in wastewater is primarily converted into nitrate (nitrification) under aerobic conditions by the combination of ammonium-oxidizing bacteria (*e.g.*, *Nitrosomonas* spp.) and nitrite-oxidizing bacteria (*e.g.*, *Nitrospira* spp.) or potentially by complete ammonia-oxidizing (comammox) bacteria (46). Nitrate is subsequently converted to N_2_ (denitrification) under anoxic conditions by diverse nitrate-reducing microorganisms. In contrast, studies on the microbiology of P removal are less comprehensive. Microbial P removal is generally performed by a group of microorganisms referred to as polyphosphate (polyP)-accumulating organisms (PAOs) (28, 53). PAOs have the ability to utilize intracellular polyP as an energy source during the anaerobic phase, during which they sequester available carbon sources. They then take up phosphate in wastewater and accumulate polyP in their cells under subsequent aerobic conditions, which results in efficient P removal from wastewater. The most well-known PAO group is *Candidatus* Accumulibacter phosphatis (hereafter called Accumulibacter spp.), classified into the family *Rhodocyclaceae* of class *Betaproteobacteria*, which has yet to be isolated in axenic cultures. Microbial community analyses based on 16S rRNA gene sequences, and also polyP staining combined with fluorescent *in situ* hybridization (FISH) and fluorescence-activated cell sorting (FACS) demonstrated that Accumulibacter spp. are the main contributors in diverse full-scale and lab-scale P-removing WWTPs (32, 52, 57, 58). In addition, a diverse range of species, including *Actinobacteria*, uncultured *Halomonadaceae*, and *Rhodocyclaceae* bacteria (not Accumulibacter spp.) have been identified as putative PAOs in full-scale SNDPR WWTPs (4, 22, 30, 31, 50). However, the paucity of pure-culture experiments has hampered furthering understanding on the ecophysiology of these microorganisms.

The oxidation ditch process is one of the most economical and efficient SNDPR techniques that simultaneously remove C, N, and P from actual sewage with repetitive aerobic/anaerobic treatment phases (7, 25, 36). Although biological C and N removal in oxidation ditch WWTPs has been extensively investigated (9, 12, 15), limited information is currently available on biological P removal. In the present study, we investigated microorganisms related to P removal in a full-scale oxidation ditch WWTP in Japan with culture-dependent and -independent approaches. Microbial community analyses based on 16S rRNA gene sequencing revealed that bacteria classified into the family *Rhodocyclaceae*, particularly *Dechloromonas* spp., predominated in the oxidation ditch WWTP. Some *Rhodocyclaceae* bacteria, including the dominant *Dechloromonas* strains, were isolated from the oxidation ditch WWTP, and their polyP accumulation abilities were examined in order to demonstrate their possible involvement in P removal from actual sewage.

## Materials and Methods

### Sample collection from the oxidation ditch WWTP

Sludge samples for the microbial community analysis and isolation of microorganisms were collected from the oxidation ditch WWTP in Okishima, Omihachiman, Shiga, Japan. This plant receives domestic wastewater with an annual average influent flow of 210 m^3^ d^−1^. The plant is generally operated with a repetitive cycle of 4-h aerobic and 6-h anaerobic phases. The aerobic (dissolved oxygen of >0.5 mg L^−1^) and anaerobic (dissolved oxygen of <0.5 mg L^−1^) phases are switched by the operation of mechanical aerators. Mixed liquor suspended solids are maintained at 5.0–8.0 g L^−1^. The annual averages of biological oxygen demand, total nitrogen, and total phosphorus in the influent/effluent are 167±93/5.0±1.2 mg L^−1^, 28.8±6.2/4.3±1.8 mg L^−1^, and 3.9±1.6/0.6±0.2 mg L^−1^, respectively, which suggests that the efficient removal of C, N, and P is achieved in the oxidation ditch WWTP. Sludge samples were recovered from the reactor during the aerobic phase.

### Microbial community and phylogenetic analyses

Microbial genomic DNA was extracted from sludge samples using the FAST DNA Spin Kit for soil (MP Biomedicals, Santa Ana, CA, USA) according to the manufacturer’s instructions. The PCR amplification of 16S rRNA gene fragments for clone library analyses was conducted as described previously with a primer set of 27F and U533R (18, 19). PCR products were purified with a QIAquick PCR Purification Kit (QIAGEN, Hilden, Germany), ligated into the pGEM-T Easy Vector (Promega, Madison, WI, USA), and cloned into *E. coli* JM109 competent cells (Promega). The sequences of the cloned PCR products were elucidated at the Biomedical Center of TAKARA Bio.

The sequences obtained were assigned to phylotypes using BLASTClust (2) with a cut-off value of 97% sequence identity. Phylotypes were phylogenetically classified with the Classifier program in the Ribosomal Database Project (47) and were compared to sequences in the GenBank nucleotide sequence database using the BLAST program (2). Phylogenetic trees were constructed using the neighbor-joining method (37) with the program MEGA7 (24). Bootstrap resampling was conducted with 1,000 replicates to validate the robustness of the phylogenetic trees. The nucleotide sequence data obtained in this study have been submitted to the GenBank database under Accession Nos. LC145217–LC145287.

### Isolation of *Rhodocyclaceae* bacteria

The isolation of *Rhodocyclaceae* bacteria was performed using an agar (1.5%)-solidified basal medium (pH 7.0) consisting of the following (L^−1^ of distilled water): 1 g of NH_4_Cl, 0.1 g of MgCl_2_·6H_2_O, 0.08 g of CaCl_2_·6H_2_O, 0.6 g of NaCl, 9.52 g of 4-(2-hydroxyethyl)-1-piperazineethanesulfonic acid, 0.1 g of yeast extract, and 10 mL each of vitamin solution and trace metal solution (19). Organic substrates (sodium acetate or sodium lactate, final concentration of 10 mM) and phosphate buffer solution (KH_2_PO_4_ and NaHPO_4_·12H_2_O, final concentration of 10 mM, pH 7.0) were separately sterilized and supplemented to the basal medium after autoclaving to avoid unintended reactions (43). Sludge samples were serially diluted and inoculated onto the medium and incubated at 25°C under aerobic conditions. After a 10-d incubation, colonies were randomly selected, and further purified with the same medium at least three times. The whole length of the 16S rRNA gene sequence was measured by direct sequencing of the DNA fragment derived from PCR amplification using the primer pair of 27F and 1492R as described previously (17).

### Microscopic observations and quantification of microbial polyP accumulation

The isolated strains and *E. coli* K-12 (as a reference strain) were cultivated to the early stationary phases in the basal media described above and supplemented with 10 mM sodium lactate. In the microscopy analysis, 1 mL of cell suspension was collected by centrifugation (8,000×*g*, 5 min), resuspended in 1 mL of phosphate-buffered saline (PBS), and stained with 4′6-diamidino-2-phenylindole (DAPI) at a final concentration of 10 μg mL^−1^ for 30 min. After staining, cells were collected by centrifugation and resuspended in PBS. PolyP fluorescence and DNA fluorescence were observed using a fluorescence microscope (DMI 4000 B, Leica) with 340–380 nm excitation and 425 LP emission filters. In the spectrofluorometric polyP analysis, 1 mL of cell suspension was stained with DAPI following the same procedure used for microscopy. After staining and centrifugation, cells were resuspended in 200 μL PBS and loaded onto a 96-well flat-bottom microplate. Cells were excited at 355 nm, polyP fluorescence was captured at 535 nm emission, and DNA fluorescence was captured at 450 nm emission using bottom fluorescence reading on a SPECTRAmax Gemini XS (Molecular Devices, Tokyo, Japan) microplate reader. In the emission scan analysis, cells were excited at 355 nm and fluorescence emission was recorded between 425 nm and 595 nm at 5-nm intervals. All excitation and emission wavelengths had a bandwidth of 9 nm. Relative polyP fluorescence values were obtained by normalizing polyP fluorescence with DNA fluorescence (21). Fluorometric experiments to monitor DNA and polyP fluorescence were conducted in triplicate and the Student’s *t*-test was used for statistical analyses.

## Results and Discussion

### Microbial community analysis of the oxidation ditch WWTP

**Fig. 1:**
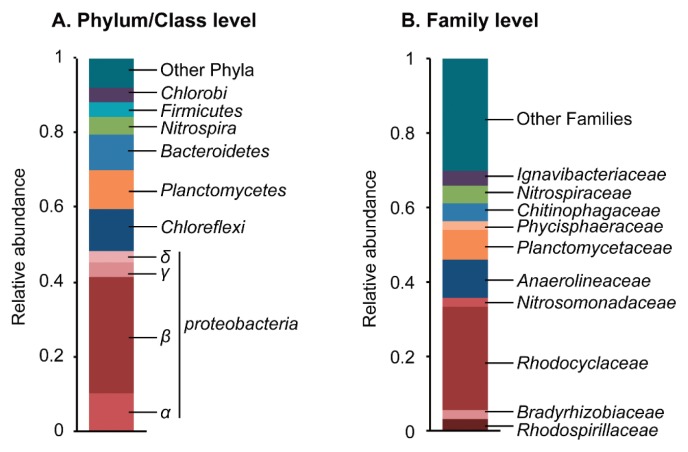
Phylogenetic distribution of 16S rRNA gene clones in the oxidation ditch WWTP. Obtained clones (total 126) were classified into a phylum/class level (A) and family level (B) and relative abundances are shown.

A clone library analysis based on the bacterial 16S rRNA gene was conducted in order to elucidate the microbial community structure in the full-scale oxidation ditch WWTP in Japan, in which the efficient removal of C, N, and P from domestic wastewater has been achieved. All phylotypes detected from the WWTP are listed in Table S1. Overall phylogenetic trends with phylum/class and family levels are shown in Fig. 1A and B, respectively. In the phylum/class level analysis (Fig. 1A), *Betaproteobacteria* (31.0% of all clones) was dominant, followed by *Chloroflexi* (11.1%), *Alphaproteobacteria* (10.3%), and *Bacteroidetes* (10.3%). The predominance of these phylogenetic groups is a common trait of SNDPR WWTPs including oxidation ditch systems (13, 15, 20, 39, 48, 49, 56).

In the family level analysis (Fig. 1B), the family *Rhodocyclaceae* (class *Betaproteobacteria*) was dominant (27.8% of all clones). *Rhodocyclaceae* bacteria are facultatively anaerobic, generally have nitrate-reducing abilities, and include some polyP-accumulating species (11, 23, 32), suggesting that *Rhodocyclaceae* bacteria contribute to the removal of C, N, and also P in the oxidation ditch WWTP. The results of further analyses on *Rhodocyclaceae* bacteria are presented in the next section.

Other dominant groups were the families *Anaerolineaceae* (phylum *Chloroflexi*) (10.3% of all clones), *Planctomycetaceae* (phylum *Planctomycetes*) (7.9%), *Chitinophagaceae* (phylum *Bacteroidetes*) (4.8%), and *Nitrospiraceae* (phylum *Nitrospira*) (4.8%). The family *Anaerolineaceae* includes strictly anaerobic bacteria that have the ability to degrade a wide range of organic compounds in various anaerobic environments, including anaerobic WWTPs (29, 51). Most members of *Planctomycetaceae* are strictly aerobic, chemoorganotrophic bacteria, and are often detected in aerobic WWTPs such as activated sludge plants (5, 41). *Chitinophagaceae* bacteria are aerobic or facultatively anaerobic, and generally have the ability to assimilate diverse sugars and amino acids (16). The family *Nitrospiraceae* as well as the family *Nitrosomonadaceae* (phylum *Betaproteobacteria*), which are aerobic nitrite- and ammonia-oxidizing bacterial groups (14), were dominant, suggesting that these bacteria contribute to the aerobic ammonia nitrification step in the oxidation ditch WWTP. Overall, the microbial community analysis revealed the co-existence of aerobic and anaerobic microorganisms in the oxidation ditch WWTP, which may be requisite for the simultaneous and efficient removal of C, N, and P.

### *Rhodocyclaceae* phylotypes detected from the oxidation ditch WWTP

**Fig. 2:**
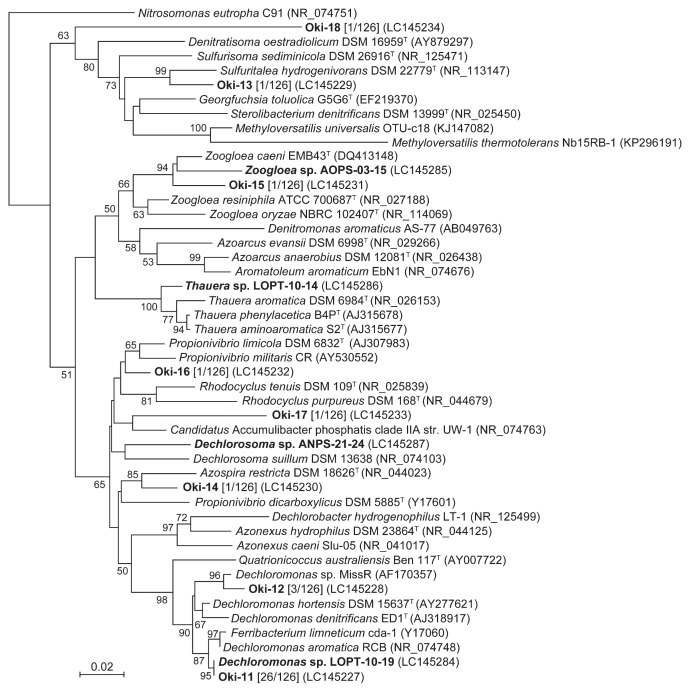
A phylogenetic tree based on partial 16S rRNA gene sequences of *Rhodocyclaceae* bacteria. The phylotypes obtained by a clone library analysis and the isolates obtained in this study are highlighted in bold letters. The numbers of clones retrieved from the clone library are shown in square brackets. Accession numbers are shown in parentheses. *Nitrosomonas eutropha* was used as an outgroup. Bootstrap values (1,000 trials, only >50% are shown) are indicated at branching points. The bar indicates 2% sequence divergence.

Since the family *Rhodocyclaceae* was the dominant bacterial group in the oxidation ditch WWTP, a detailed phylogenetic analysis was performed on the *Rhodocyclaceae* phylotypes. The phylogenetic positions of the *Rhodocyclaceae* phylotypes detected in the present study were analyzed by comparisons with sequences of representative *Rhodocyclaceae* strains in the databases (Fig. 2). The first and second most dominant phylotypes, Oki-11 and Oki-12 (20.6 and 2.4% of all clones, respectively), were closely related to *Dechloromonas* spp. (99.0% sequence identity to *Dechloromonas aromatica* strain RCB and 98.1% to *Dechloromonas* sp. MissR, respectively). *Dechloromonas* spp. are facultatively anaerobic, nitrate-reducing bacteria, and were originally isolated as aromatic compound-degrading bacteria (6). *Dechloromonas* spp. have frequently been detected in aerobic/anaerobic WWTPs (13, 20, 39, 49, 56), suggesting their contribution to the degradation of organic compounds under aerobic and anaerobic conditions and also to N removal via nitrate reduction. Furthermore, *Dechloromonas* spp. have been detected in P-removing WWTPs (10, 26, 45), suggesting their potential contribution to P removal. However, it remains controversial whether *Dechloromonas* spp. actually function as PAOs. The results of the genome analysis showed that *D. aromatica* strain RCB harbored genes required for polyP accumulation, including polyphosphate kinase and exopolyphosphatase (38). Furthermore, polyP staining coupled with FISH (23) and FACS (11) revealed that *Dechloromonas* spp. have the ability to store polyP in full-scale WWTPs. In contrast, some research groups reported opposite findings (1, 27, 40); *Dechloromonas*-related bacteria that dominated in P-removal WWTPs did not accumulate polyP *in situ*. Furthermore, there have been no studies on the polyP-accumulating abilities of *Dechloromonas* spp. using pure-culture experiments with isolated strains.

The other *Rhodocyclaceae* phylotypes (Oki-13 to -18, Fig. 2 and Supplementary Table S1) were also detected in the oxidation ditch WWTP. It is important to note that two phylotypes (Oki-16 and -17) are related to *Candidatus* Accumulibacter phosphatis (sequence identities of 96.9 and 93.0%, respectively), suggesting their ability to accumulate polyP. However, these phylotypes only comprise a minor part of the population, indicating that their contribution to P removal in the oxidation ditch WWTP is marginal.

### Isolation of *Rhodocyclaceae* bacteria from the oxidation ditch WWTP

The isolation of *Rhodocyclaceae* bacteria (particularly the dominant *Dechloromonas* phylotypes) was undertaken in order to investigate their physiology using pure-culture experiments. Based on previous findings showing that most *Rhodocyclaceae* bacteria prefer short chain organic acids as growth substrates (32), media supplemented with acetate or lactate were utilized for isolation. Colonies appearing on plate media were purified, and the partial sequences of the 16S rRNA gene were obtained. Among the 290 isolates tested, 121 isolates were classified into the family *Rhodocyclaceae*. Among them, 43 isolates were closely related to *Dechloromonas* spp. with 98–100% sequence identities, and some of them showed identical sequences to the dominant phylotype Oki-11 (*e.g.*, the isolate LOPT-10–19 in Fig. 2). Furthermore, some isolates closely related to the other genera of *Rhodocyclaceae* were also obtained. Four representative *Rhodocyclaceae* isolates classified into different genera were subjected to further phylogenetic analyses by sequencing the whole length of the 16S rRNA gene (Fig. 2). These four strains, namely *Dechloromonas* sp. LOPT-10–19, *Zoogloea* sp. AOPS-03–15, *Thauera* sp. LOPT-10–14, and *Dechlorosoma* sp. ANPS-21–24, were subjected to physiological analyses.

### PolyP-accumulating abilities of *Rhodocyclaceae* isolates

**Fig. 3:**
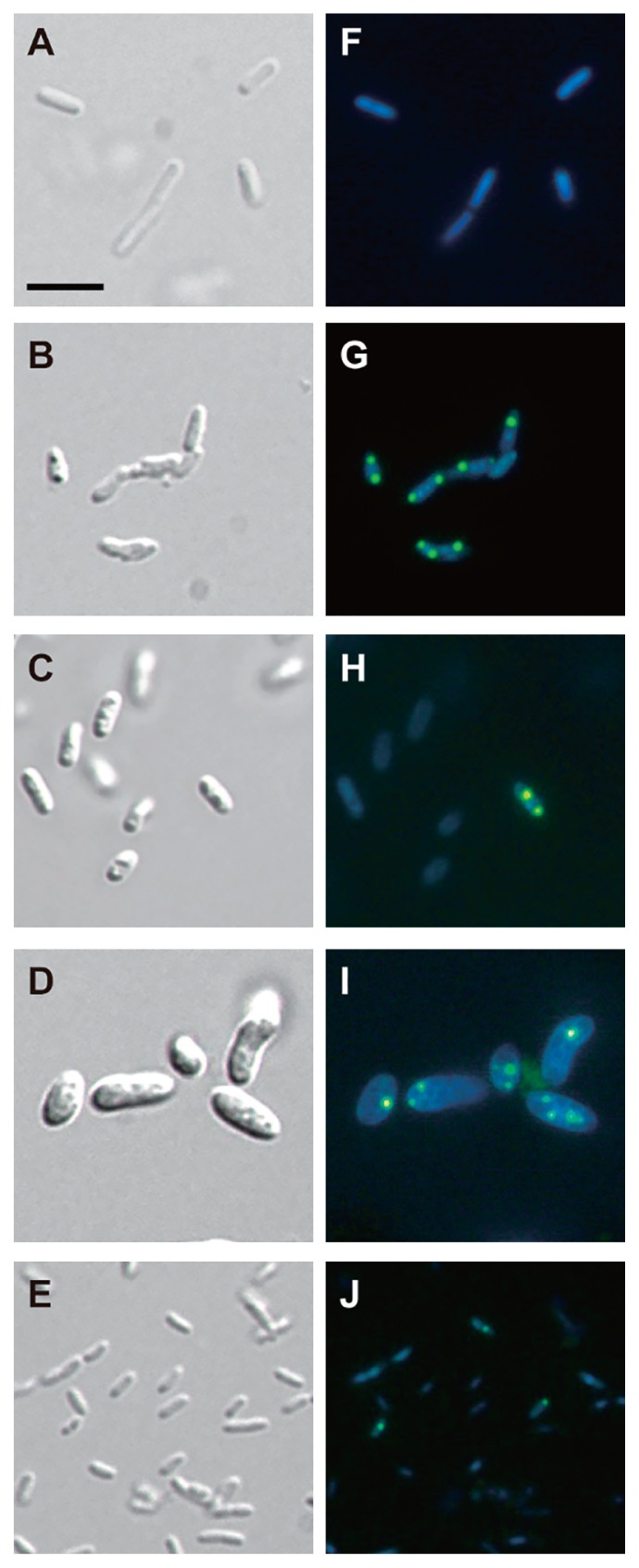
Microscopic observation of polyP accumulation by *Rhodocyclaceae* isolates. (A and F) *E. coli*, (B and G) *Dechloromonas* sp. LOPT-10–19, (C and H) *Zoogloea* sp. AOPS-03–15, (D and I) *Thauera* sp. LOPT-10–14, and (E and J) *Dechlorosoma* sp. ANPS-21–24 were stained with DAPI and microscopically observed. The bright field images (A–E) and fluorescence microscopy images (F–J) of identical microscopic fields for each strain are shown. In fluorescence microscopy images, DNA (blue) and polyP (yellow) were simultaneously observed. The scale bar represents 5 μm for all images.

The ability to accumulate polyP by *Rhodocyclaceae* isolates was assessed through fluorescence staining approaches. DAPI is a fluorescent chemical that is widely used to visualize polyP stored in microbial cells (42, 44). DAPI stains DNA in addition to polyP, but with distinct fluorescence emission (blue and yellow-green, respectively) when excited with UV light (3, 33). The representative *Rhodocyclaceae* isolates and *E. coli* were aerobically cultivated in basal medium supplemented with lactate. Cells in the stationary phases were collected, stained with DAPI, and subjected to fluorescence microscopy (Fig. 3). Yellow-green fluorescence was not observed in DAPI-stained *E. coli* cells (Fig. 3F). In contrast, almost all cells of *Dechloromonas* sp. LOPT-10–19 exhibited yellow-green fluorescence derived from polyP granules (Fig. 3G). A previous study reported that the numbers and cellular localization of polyP granules differ between PAOs, ranging from a single large granule at one end of a cell to multiple granules scattered within a cell (8). The accumulation of polyP at polar ends of the rod-shaped cell in *Dechloromonas* sp. LOPT-10–19 is very similar to the accumulation patterns observed for *Corynebacterium* (34); however, the mechanisms underlying particular accumulation patterns are currently unknown. The other *Rhodocyclaceae* isolates also exhibited yellow-green fluorescence, while the manner of polyP accumulation was dissimilar to that of *Dechloromonas* sp. LOPT-10–19. Regarding *Zoogloea* sp. AOPS-03–15 and *Dechlorosoma* sp. ANPS-21–24, only minor fractions of cells exhibited yellow-green fluorescence (Fig. 3H and J). As for *Thauera* sp. LOPT-10–14 (Fig. 3I), while most cells exhibited yellow-green fluorescence, the intensity and size of polyP granules relative to the cell area appeared to be smaller than those of *Dechloromonas* sp. LOPT-10–19. *Thauera* sp. LOPT-10–14 has a larger cell size (~3–5 μm in length and ~2 μm in width) than *Dechloromonas* sp. LOPT-10–19 (~1.5–3 μm in length and ~0.8 μm in width). However, polyP granules were of a similar size for both strains (~0.5–0.8 μm in diameter).

**Fig. 4:**
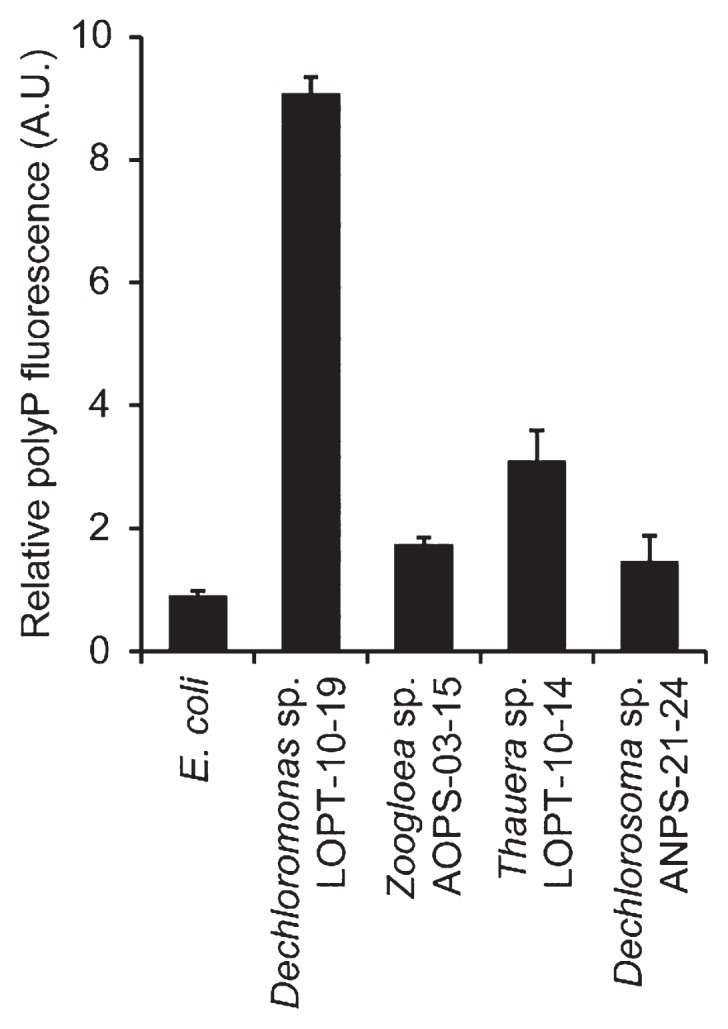
Fluorescence quantification of polyP accumulation by *Rhodocyclaceae* isolates. The cell suspension was excited by ultraviolet light of 355 nm, and fluorescence derived from polyP (535 nm emission) was normalized with that derived from DNA (450 nm emission). Values are presented as the means of three independent cultures, and error bars indicate standard deviations.

In order to quantitatively compare polyP-accumulating activities, DAPI-stained cell suspensions were subjected to spectrofluorometry. The relative amounts of polyP stored in cells were assessed by normalizing polyP-derived fluorescence (535 nm emission) using DNA-derived fluorescence (450 nm emission) (Fig. 4 and Supplementary Fig. S1). Relative polyP fluorescence from *Dechloromonas* sp. LOPT-10–19 was more than 10-fold higher than the background levels observed in the reference strain (*E. coli* K-12). The other *Rhodococcaceae* strains also exhibited significantly higher polyP fluorescence than *E. coli*; however, it was still markedly less than that of *Dechloromonas* sp. LOPT-10–19. These results were consistent with the microscopy images (Fig. 3), in which *Zoogloea* sp. AOPS-03–15 and *Dechlorosoma* sp. ANPS-21–24 both had fewer polyP granules than *Dechloromonas* sp. LOPT-10–19, and, as discussed above, *Thauera* sp. LOPT-10–14 had larger cells, making relative polyP fluorescence lower. The results of these microscopic and fluorometric analyses demonstrated that *Dechloromonas* spp. dominantly detected in the oxidation ditch WWTP have strong abilities to intracellularly accumulate polyP. This study is the first to demonstrate significant polyP accumulation by *Dechloromonas* spp. in pure-culture experiments, and provides an insight into the contribution of *Dechloromonas* spp. to microbial P removal in diverse WWTPs. The future challenge is to understand the role of *Dechloromonas* spp. in P removal in the WWTP, *e.g.*, collecting polyP-accumulating cells from *in situ* microbial communities using DAPI staining combined with flow cytometry, followed by phylogenetic identification. By carefully following polyP accumulation in *Dechloromonas* spp. in the WWTP, P removal activity within the greater microbial community may be more clearly understood.

## Conclusion

In the present study, we demonstrated the dominance of *Rhodocyclaceae* bacteria, particularly *Dechloromonas* spp., in the full-scale oxidation ditch WWTP in Japan, in which C, N, and also P are stably and efficiently removed. Some strains closely related to *Dechloromonas* spp. were successfully isolated and proven to exhibit strong abilities to intracellularly accumulate polyP. This is the first study to demonstrate polyP accumulation by *Dechloromonas* spp. in pure-culture experiments. These results suggest that *Dechloromonas* spp. is an important player in the removal of C, N, and also P in the oxidation ditch WWTP.

## Supplementary Information
