# Supplementary material for: Culture-Dependent and -Independent Identification of Polyphosphate-Accumulating Dechloromonas spp. Predominating in a Full-Scale Oxidation Ditch Wastewater Treatment Plant
Source: Microbes Environ. 2016 Nov 19;31(4):449–55. doi: 10.1264/jsme2.ME16097 (PMC5158118; doi:10.1264/jsme2.ME16097)
Supplement: Supplementary file 1 [file 31_449_s1.pdf]

**Supplemental Table S1.** The phylotypes detected from the oxidation ditch WWTP.

| Phylotype | Phylogenetic group | | Closest relative (identity, %) | Number of clones |
|---|---|---|---|---|
| | Phylum/Class | Family | | |
| Oki-01 | *Alfaproteobacteria* | *Bradyrhizobiaceae* | KC247114 *Bradyrhizobium* sp. CMVU04 (95.8) | 3 |
| Oki-02 | | *Rhodospirillaceae* | FJ455532 *Dongia mobilis* strain LM22 (91.3) | 2 |
| Oki-03 | | | HM748667 Bacterium Ellin6519 (87.2) | 1 |
| Oki-04 | | | AB245345 Alpha proteobacterium Gsoil 264 (90.4) | 1 |
| Oki-05 | | *Hyphomicrobiaceae* | JX392051 *Devosia* sp. DDB001 (96.1) | 1 |
| Oki-06 | | | NR_074324 *Nitrobacter winogradskyi* Nb-255 (95.8) | 1 |
| Oki-07 | | *Phyllobacteriaceae* | AB289614 *Mesorhizobium loti* strain NGT514 (98.7) | 1 |
| Oki-08 | | | KC146415 *Ochrobactrum* sp. DZQ2a (92.7) | 1 |
| Oki-09 | | *Rickettsiaceae* | AB698680 *Methylobacterium* sp. 92d (86.6) | 1 |
| Oki-10 | | *Rhodobacteraceae* | Y09610 *Tetracoccus cechii* (93.7) | 1 |
| Oki-11 | *Betaproteobacteria* | *Rhodocyclaceae* | NR_074748 *Dechloromonas aromatica* RCB (99.0) | 26 |
| Oki-12 | | | AF170357 *Dechloromonas* sp. MissR (98.1) | 3 |
| Oki-13 | | | AB552842 *Sulfuritalea hydrogenivorans* (95.2) | 1 |
| Oki-14 | | | NR_024855 *Propionivibrio pelophilus* strain asp 66 (95.7) | 1 |
| Oki-15 | | | DQ413157 *Zoogloea* sp. EMB 108 (96.0) | 1 |
| Oki-16 | | | NR_074763 *Candidatus* Accumulibacter phosphatis clade IIA str. UW-1 (96.9) | 1 |
| Oki-17 | | | NR_074763 *Candidatus* Accumulibacter phosphatis clade IIA str. UW-1 (93.0) | 1 |
| Oki-18 | | | NR_043249 *Denitratisoma oestradiolicum* strain AcBE2-1 (87.8) | 1 |
| Oki-19 | | *Nitrosomonadaceae* | CP002876 *Nitrosomonas* sp. Is79A3 (95.1) | 3 |
| Oki-20 | | *Comamonadaceae* | GU368379 *Curvibacter lanceolatus* strain JPPB B27 (95.6) | 1 |

**Table S1.** Continued.

| Phylotype | Phylogenetic group: Phylum/Class | Family | Closest relative (identity, %) | Number of clones |
|---|---|---|---|---|
| Oki-21 | *Gammaproteobacteria* | *Moraxellaceae* | HE582776 *Agitococcus lubricus* strain DSM 5822T (93.5) | 1 |
| Oki-22 | | | JQ782897 *Acinetobacter* sp. zol-02 (99.2) | 1 |
| Oki-23 | | *Methylococcaceae* | HQ433439 *Halomonas* sp. E2B(2010) (86.3) | 1 |
| Oki-24 | | *Sinobacteraceae* | AB819626 *Pseudomonas* sp. VT1B (92.9) | 1 |
| Oki-25 | | *Pseudomonadaceae* | HM590828 *Azonexus* sp. HME6654 (98.6) | 1 |
| Oki-26 | *Deltaproteobacteria* | *Syntrophaceae* | NR_074968 *Anaeromyxobacter* sp. Fw109-5 (82.5) | 1 |
| Oki-27 | | | AY835391 *Desulfuromonas svalbardensis* strain 103 (85.0) | 1 |
| Oki-28 | | *Cystobacteraceae* | AM930267 *Melittangium lichenicola* strain DSM 14877 (89.6) | 1 |
| Oki-29 | | *Nannocystaceae* | FR749907 *Nannocystis pusilla* strain DSM 14622T (90.8) | 1 |
| Oki-30 | *Chloroflexi* | *Anaerolineaceae* | NR_074383 *Anaerolinea thermophila* UNI-1 (90.0) | 6 |
| Oki-31 | | | AB112774 Filamentous symbiotic bacterium of *Methylobacterium* sp. strain TFF (96.7) | 6 |
| Oki-32 | | | NR_040972 *Levilinea saccharolytica* strain KIBI-1 (87.9) | 1 |
| Oki-33 | | *Caldilineaceae* | NR_074397 *Caldilinea aerophila* DSM 14535 (88.2) | 1 |
| Oki-34 | *Planctomycetes* | *Planctomycetaceae* | FJ405890 *Planctomycetacia* bacterium WSF3-27 (88.0) | 3 |
| Oki-35 | | | JF488160 *Planctomycetes* bacterium SCGC AAA204-A13 (96.7) | 3 |
| Oki-36 | | | AF239694 *Gemmata*-like str. JW3-8s0 (83.2) | 2 |
| Oki-37 | | | NR_025327 *Planctomyces maris* strain 534-30 (81.1) | 1 |
| Oki-38 | | | X81947 *Pirellula* sp. Schlesner 678 (95.5) | 1 |
| Oki-39 | | *Phycisphaeraceae* | AY162122 *Planctomycete* GMD14H10 (83.3) | 2 |
| Oki-40 | | | AY162122 *Planctomycete* GMD14H10 (85.8) | 1 |

**Table S1.** Continued.

| Phylotype | Phylogenetic group | | Closest relative (identity, %) | Number of clones |
|---|---|---|---|---|
| | Phylum/Class | Family | | |
| Oki-41 | *Bacteroidetes* | *Chitinophagaceae* | NR_044588 *Ferruginibacter alkalilentus* strain HU1-GD23 (93.3) | 2 |
| Oki-42 | | | KF228170 *Lacibacter cauensis* clone B10-0AF4I_11038 (82.0) | 1 |
| Oki-43 | | | FN396961 *Flexibacter* sp. AMV16 (80.8) | 1 |
| Oki-44 | | | JF834159 *Terrimonas* sp. QH (87.8) | 1 |
| Oki-45 | | | NR_041639 *Filimonas lacunae* strain YT21 (82.5) | 1 |
| Oki-46 | | *Cytophagaceae* | KC560020 *Mucilaginibacter sp. RG4-7* (84.2) | 1 |
| Oki-47 | | | NR_102866 *Cytophaga hutchinsonii* ATCC 33406 (96.7) | 1 |
| Oki-48 | | *Cryomorphaceae* | FJ424814 *Wandonia haliotis* strain Haldis-1 (96.7) | 2 |
| Oki-49 | | *Saprospiraceae* | AJ431246 *Dehalococcoides* sp. BHI80-15 (81.1) | 1 |
| Oki-50 | | unassigned | AB308367 Bacterium TG141 (94.4) | 1 |
| Oki-51 | *Nitrospira* | *Nitrospiraceae* | GQ249372 *Candidatus* Nitrospira defluvii clone B14 (98.9) | 4 |
| Oki-52 | | | Y14644 *Nitrospira* sp. strain GC86 (98.7) | 2 |
| Oki-53 | *Firmicutes* | *Pasteuriaceae* | NR_074521 *Pirellula staleyi* DSM 6068 (86.7) | 2 |
| Oki-54 | | *Lactobacillaceae* | JQ805635 *Lactobacillus gasseri* strain IMAUFB015 (99.5) | 1 |
| Oki-55 | | *Erysipelotrichaceae* | GU470893 *Solobacterium moorei* strain F0204 (90.2) | 1 |
| Oki-56 | | *Lachnospiraceae* | HF549104 *Heliobacillus* sp. JHB29 (81.5) | 1 |
| Oki-57 | *Chlorobi* | *Ignavibacteriaceae* | NR_074698 *Ignavibacterium album* JCM 16511 (91.8) | 5 |
| Oki-58 | *Actinobacteria* | *Acidimicrobineae* | AB517669 *Aciditerrimonas ferrireducens* (91.3) | 1 |
| Oki-59 | | *Intrasporangiaceae* | NR_042621 *Terrabacter lapilli* strain LR-26 (93.1) | 1 |
| Oki-60 | | *Propionibacterineae* | AB021325 Bacterium rJ7 (93.1) | 1 |

**Table S1.** Continued.

| Phylotype | Phylogenetic group | | Closest relative (identity, %) | Number of clones |
|---|---|---|---|---|
| | Phylum/Class | Family | | |
| Oki-61 | OD1 | unassigned | HQ132472 Uncultured bacterium clone X-50 (97.2) | 1 |
| Oki-62 | | | AY193203 Uncultured candidate division OD1 bacterium clone DA35 (86.2) | 1 |
| Oki-63 | | | GU505743 Uncultured bacterium clone F1Q32TO06HNCMU (97.8) | 1 |
| Oki-64 | *Verrucomicrobia* | *Verrucomicrobiaceae* | KF228173 *Prosthecobacter dejongeii* clone F11-0AF4F_11038 (93.4) | 1 |
| Oki-65 | | unassigned | AY960781 Bacterium Ellin518 (90.8) | 1 |
| Oki-66 | *Lentisphaerae* | *Lentisphaeraceae* | AB498915 *Lentisphaerae* bacterium 7509 (84.3) | 1 |
| Oki-67 | TM7 | unassigned | CP005957 *Candidatus* Saccharimonas aalborgensis (88.6) | 1 |
| Total | | | | 126 |

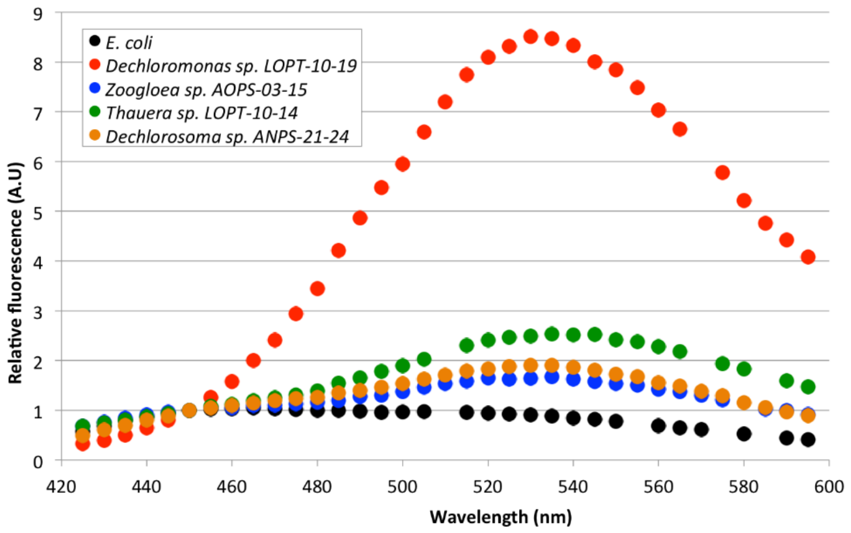


**Supplementary Fig. S1.** Fluorescence emission scan of DAPI-stained *E. coli* and *Rhodocyclaceae* isolates excited at 355 nm. Fluorescence emission was measured from 425 nm to 595 nm and normalized to DNA emission peak of *E. coli* cells (450 nm).
